# Quality improvement collaboratives as part of a quality improvement intervention package for preterm births at sub-national level in East Africa: a multi-method analysis

**DOI:** 10.1136/bmjoq-2023-002443

**Published:** 2023-12-22

**Authors:** Rogers Mandu, Lara Miller, Gertrude Namazzi, Nana Twum-Danso, Kevin Jacton Abidha Achola, Isabella Cooney, Elizabeth Butrick, Nicole Santos, Leakey Masavah, Alphonce Nyakech, Leah Kirumbi, Peter Waiswa, Dilys Walker

**Affiliations:** 1 School of Public Health, Makerere University, Kampala, Kampala, Uganda; 2 Institute for Global Health Sciences, University of California San Francisco, San Francisco, California, USA; 3 Independent Consultant, New York, New York, USA; 4 Kenya Medical Research Institute, Nairobi, Kenya; 5 Karolinska Institutet, Stockholm, Stockholm, Sweden; 6 Department of Obstetrics and Gynecology and Global Health Sciences, University of California San Francisco Medical Center at Parnassus, San Francisco, California, USA

**Keywords:** Paediatrics, Health services research, Implementation science, Maternal Health Services, PDSA

## Abstract

**Background:**

Quality improvement collaboratives (QIC) are an approach to accelerate the spread and impact of evidence-based interventions across health facilities, which are found to be particularly successful when combined with other interventions such as clinical skills training. We implemented a QIC as part of a quality improvement intervention package designed to improve newborn survival in Kenya and Uganda. We use a multi-method approach to describe how a QIC was used as part of an overall improvement effort and describe specific changes measured and participant perceptions of the QIC.

**Methods:**

We examined QIC-aggregated run charts on three shared indicators related to uptake of evidence-based practices over time and conducted key informant interviews to understand participants’ perceptions of quality improvement practice. Run charts were evaluated for change from baseline medians. Interviews were analysed using framework analysis.

**Results:**

Run charts for all indicators reflected an increase in evidence-based practices across both countries. In Uganda, pre-QIC median gestational age (GA) recording of 44% improved to 86%, while Kangaroo Mother Care (KMC) initiation went from 51% to 96% and appropriate antenatal corticosteroid (ACS) use increased from 17% to 74%. In Kenya, these indicators went from 82% to 96%, 4% to 74% and 4% to 57%, respectively. Qualitative results indicate that participants appreciated the experience of working with data, and the friendly competition of the QIC was motivating. The participants reported integration of the QIC with other interventions of the package as a benefit.

**Conclusions:**

In a QIC that demonstrated increased evidence-based practices, QIC participants point to data use, friendly competition and package integration as the drivers of success, despite challenges common to these settings such as health worker and resource shortages.

**Trial registration number:**

NCT03112018.

**WHAT IS ALREADY KNOWN ON THIS TOPIC**

- Quality improvement (QI) efforts led by facility-based teams are an important tool for improving quality of care. Collaboration across QI teams from multiple facilities in a QI collaborative can facilitate peer learning, though results on health outcomes have been mixed.

**WHAT THIS STUDY ADDS**

- Our study describes a QI collaborative that was introduced in Kenya and Uganda alongside clinical and teamwork skills training, data strengthening and the WHO Safe Childbirth Checklist—a quality improvement package that resulted in improved neonatal outcomes. We show the improvement of shared QI indicators, as well as illustrate health workers’ appreciation for cross-facility learning, friendly competition and the integration of QI practices with other interventions.

**HOW THIS STUDY MIGHT AFFECT RESEARCH, PRACTICE OR POLICY**

- This study highlights the importance of QI efforts in multi-component interventions and provides insight into the added value of working across facilities using a QI Collaborative approach.

## Introduction

Every year, an estimated 1.8 million children die in the first month of life. Of these, 98% are in low-income and middle-income countries (LMICs).^1^ Neonatal mortality has stagnated in both Kenya and Uganda at 21 and 20 deaths per 1000 live births, respectively.^2^ The regional average absolute risk reduction in neonatal mortality was only 1.9% from 1990 to 2017 as the facility birth rate increased from an average of 38% to 67%.^2–4^ This plateau, despite continued increases in facility-based birth, indicates that additional attention to the quality of care during the intrapartum and early newborn period is needed.

Routine and effective implementation of low-cost interventions could avert an estimated 71% of the neonatal deaths in LMICs.^5^ As 44% of all these neonatal deaths occur within the first 24 hours of life^6^ and an additional 2.6 million babies are stillborn,^7^ targeting the intrapartum and immediate postnatal period when infants are most likely to be in health facilities is a critical window of opportunity for improvement. Furthermore, about one-third of neonatal deaths are associated with complications due to prematurity.^8^ Improving quality of care—particularly for the most fragile babies who are born sick, small or early—will most likely improve newborn survival.^9^

The biggest gains can be made by enhancing uptake of evidence-based practices—standard clinical practices that have been demonstrated to improve outcomes in rigorous research.^10^ Both Kenya and Uganda have national policies in place that support the organisation of frontline workers into quality improvement teams (QITs) that conduct iterative improvement cycles.^11 12^ The QIT approach posits that frontline workers can identify and solve barriers to quality if given analytical tools and supported by leadership to make local institutional and workflow changes. Further, platforms for peer learning and group problem solving across a network of facilities such as quality improvement collaboratives (QICs) have been shown to increase the adoption of improvement ideas and practices and accelerate overall improvement.^13–17^ While QICs have demonstrated success in increasing use of evidence-based practices in high-income settings,^16–18^ a systematic review found the evidence to be generally positive, but inconsistent and limited.^19^ Another meta-analysis that focused on QICs in LMICs similarly found limited evidence but concluded that QICs paired with provider clinical training were more effective than QICs alone.^20^

In 2015, the Preterm Birth Initiative East Africa (PTBi-EA) set out to reduce the burden of prematurity in Kenya and Uganda by focusing on improving quality of care, targeting small and sick newborns. We implemented a four-part quality improvement (QI) intervention package wherein all components reinforced the uptake of known evidence-based practices.^21^ All sites (intervention and control) received data strengthening and a modified Safe Childbirth Checklist (mSCC) to improve recognition of prematurity and reinforce data documentation and compliance to care protocols. Intervention sites received two additional interventions: provider clinical and teamwork training using the PRONTO curriculum and country-specific QICs. While PRONTO simulation and team training addressed provider clinical knowledge, skills and teamwork, and identified workflow and system gaps, QICs addressed these issues through systems analyses and testing and iteration of local solutions by frontline providers and their managers. Staff delivering and receiving these two interventions overlapped and the elements complemented each other. Reports on our implementation as an integrated package and additional detail about other interventions separately are available elsewhere.^22–24^

The results of the cluster randomised trial evaluating the PTBi-EA QI package are reported elsewhere; in short, we observed a significantly reduced odds of neonatal death at intervention facilities compared with control sites (OR 0.66, 95% CI 0.54–0.81).^25^ While the package components were integrated, closer examination of each is helpful to understand implementation and how integration may have contributed to impact. The aim of this paper is to describe the use of QICs in the PTBi-EA package, document the progress made in those QICs and describe healthcare provider experiences to gain understanding of how this component may have contributed to the overall study outcomes.

## Methods

### Study design

We used a multi-method approach to understand the impact of QICs as part of the PTBi-EA QI package. To determine the impact of QICs, we analysed aggregated data for key process indicators of evidence-based practices implemented by QI teams across study sites. We explored perceptions and perceived impact among QI team members through in-depth interviews (IDIs).

### Setting

The PTBi-EA package was implemented in Migori County in western Kenya and the Busoga Region of eastern Uganda. The project included 23 health facilities, of which 20 were pair matched and randomised, as described elsewhere.^21^ Three referral facilities were excluded from randomisation but included in the intervention to ensure improvements in quality of care across the full continuum of care for ethical reasons. Facilities included six district hospitals in Uganda (four public, two not-for-profit missionary) and one county and 12 subcounty hospitals plus four health centres in Kenya (15 public facilities, two not-for profit missionary hospitals). The QICs included the two intervention-randomised facilities plus two referral hospitals in Uganda and eight intervention-randomised facilities plus one referral hospital in Kenya.

### QIC intervention description

Prior to implementation, study leadership in Kenya, Uganda and USA, together with the administrative health leaders of Busoga Region and Migori County, agreed on core process indicators to be tracked that reflected key evidence-based practices most likely to result in improvement of clinical outcomes among preterm neonates. These included gestational age (GA) determination, use of antenatal corticosteroids (ACS) for eligible women in labour and uptake of immediate Kangaroo Mother Care (KMC) for eligible babies.

Study QI mentors who were specialists in obstetrics and gynaecology, paediatricians, medical officers, midwives or nurses with additional training in QI methods were identified, with 1–2 QI mentors plus 4–8 clinical mentors with QI training per country. QI mentors and PTBi study staff were trained and coached longitudinally by an international consultant (NT-D) with experience in large QICs focused on maternal and child health. QI mentors visited intervention facilities, collected baseline data for the indicators and helped each facility form a QIT. QITs comprised doctors, midwives, nurses and clinical officers who served the maternity and newborn care units in that facility. Each team had 6–12 members who would meet every 1–2 weeks to work on their improvement goals related to the focus of the PTBi-EA.

Throughout the study, QI mentors visited facilities to follow-up with QITs, first weekly, then biweekly and tapering to monthly by project end as designed for the purposes of sustainability and institutionalisation. During these visits, mentors worked with QITs on identifying system gaps, inefficiencies in processes, root cause analyses, development of change ideas/solutions, testing of the solutions and evaluation through the Plan, Do, Study, Act (PDSA) cycle. Teams subsequently implemented the change ideas that were assessed to be successful. Common themes included workflow refinements or process redesigns, reminder systems, re-trainings and communication strategies to keep the care team aligned. QITs could apply for and receive catalytic funds for improvement projects that required discrete one-time inputs to solve identified bottlenecks, not exceeding US$250. A list of change ideas generated can be found in online supplemental table S1.

10.1136/bmjoq-2023-002443.supp1 Supplementary data

**Figure 1:**
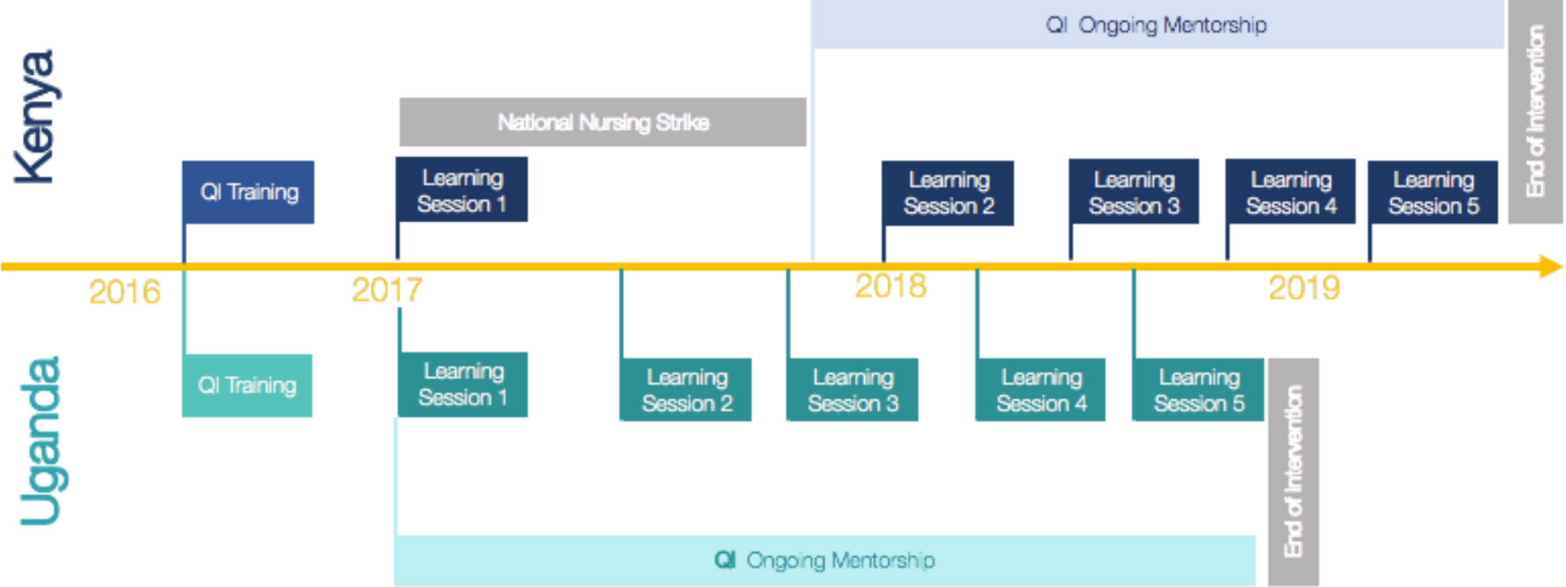
Timeline.

Each country’s QIC met in a series of five learning sessions, spaced 3–6 months apart over the project period (figure 1). Participants included two to three rotating team members from each facility. Led by the QI consultant, each learning session included sharing of progress by each facility regarding the agreed process indicators and change ideas being tested, and introduction or review of QI methods to strengthen QI capacity in the teams. In each session, QITs shared their progress in applying the key evidence-based practices through run charts, discussed their approaches to improving them and saw how they compared with other facilities. Participants used QI methods to interpret each QIT’s data and help plan for further improvement. Breakout sessions during learning sessions explicitly facilitated peer-to-peer learning and sharing through ‘Marketplace’ sessions or storyboards across QITs/facilities as well as real-time application of the QI methods they were learning to their specific context.

After the first two learning sessions, additional time was added to each learning session to ensure county/district leadership attended part of the session to hear about their teams’ progress and challenges, support problem solving and better appreciate their role as leaders in enabling sustained improvement in their institutions. Contact with QI mentors and colleagues at other facilities in the learning sessions led to the development of natural professional support networks, including, for example, a WhatsApp group organised among participants for problem solving related to maternal newborn health issues.

The QIC in Kenya was impacted by a protracted health worker strike (see figure 1), resulting in the second learning session serving as a refresher of the content taught in the first learning session and a reset of the QIC. On the contrary, the Uganda QIC completed activities as originally scheduled and was able to implement a sustainability plan including holding two additional learning sessions in which successful change ideas and key QI methods were shared with PTBi-EA study facilities in the control arm.

Throughout the life of the project, we worked to ensure sustainability in several ways. First, the role and engagement of QI coaches diminished over time—they began by leading teams and decreased their engagement over time, letting teams lead themselves as they gained competence. Second, the role of the county/district and hospital leadership in the QIC increased over time through engagement during learning sessions as well as during the activity period. Third, each learning session continued to build capacity by adding additional QI methods or reviewing previously taught ones. Interaction with teams across facilities was done in QIC learning sessions, but also facilitated thorough WhatsApp groups, establishing open communication channels that easily outlived the project. Additionally, each QIC was charged with sharing their successful change ideas and QI methods with surrounding facilities not involved in the PTBi-EA project, although only Uganda did this during the original project period. Lastly, while QIC meeting costs and transport were paid by the PTBi project, QIT members received no financial incentives.

### Patient and public involvement

Patients were not directly involved in the development of research questions, outcome measures, study design or execution. The QI intervention specifically targeted healthcare providers and randomisation was at the level of the health facility. All data were aggregated with no personal identifiers collected. Local health authorities were consulted and included in implementation.

### Data collection

Quantitative QI data for run charts were collected by QI mentors and QITs monthly during mentor visits during the study period. Data were collected on three core process indicators (table 1) across all teams in both QICs—GA determination, use of ACS for eligible women and uptake of KMC for eligible babies.

**Table 1:** Indicator definitions

Process indicators				
Indicator	Numerator	Data source Numerator	Denominator	Data source Denominator
Antenatal corticosteroid (ACS) administration	Number of admitted women with GA <34 weeks presenting with an increased risk of preterm delivery within the next 7 days who received ACS. Mother must be free of contraindications including systemic infection (including TB and/or sepsis) and immunosuppression (including untreated HIV).	Maternity patient charts and/or modified Safe Childbirth Checklist	Number of admitted women with GA <34 weeks presenting with an increased risk of preterm delivery within the next 7 days	Maternity patient charts and/or modified Safe Childbirth Checklist
Gestational age (GA) documentation	Number of maternity admissions with documented GA.	Maternity Register	Number of women admitted into the maternity ward	Maternity Register
Kangaroo Mother Care (KMC) initiated	Number of mothers/caregivers with eligible infants born <2.5 kg counselled on and initiating KMC prior to discharge. Eligible infants are defined as clinically stable infants born <2.5 kg in the facility that have no signs of respiratory distress and no signs of severe infection. Signs of respiratory distress include nasal flaring, respiratory rate >60 or <40, apnoea, grunting respiration, chest in-drawing, cyanosis. Signs of severe infection include distended abdomen.	Sick Newborn Register and/or Newborn Patient Charts	Number of mothers that deliver eligible (clinically stable) newborns born <2.5 kg in the facility	Maternity patient charts

TB, tuberculosis.

Qualitative QI data were collected through IDIs with PTBi-EA stakeholders as part of process or endline evaluations. We examined a subset of these IDIs, from health workers, nursing officers and facility in-charges from intervention facilities. Interviewees were selected purposively based on their length of employment in the maternity ward and their involvement in the QI activities. Data included 11 IDIs from seven intervention facilities in Kenya and 16 IDIs from four intervention sites (including the non-randomised referral facilities) in Uganda. IDIs explored perceptions, acceptability and sustainability of the QI work. Interviews were conducted by trained research assistants using a structured interview guide written to collect data on the entirety of the PTBi project.

### Data management

Data on shared indicators were collected by the QITs and the QI mentors from maternal and newborn data sources including mSCC forms, maternity patient charts where GA estimation and ACS provision were documented and the sick newborn registers where KMC initiation was documented. Indicator tallies were hand recorded and plotted on run charts by the QITs and then transferred by the QI mentors to an Open Data Kit form hosted on a server at UCSF. QITs also used a documentation journal for each of the indicators selected in which tested change ideas were documented.

All qualitative interviews were audio recoded and notes taken during the interviews. All audio recordings were transcribed verbatim by experienced research assistants. All data and materials were kept under lock and key by the study managers in the respective countries. Transcripts were stored online and analysed using a secure Box server.

### Data analysis

Quantitative data across individual facility run charts were aggregated for each country QIC to show overall trends. Prior to the first learning session, data from 3 months were collected to serve as a baseline, and the median baseline value is plotted on each run chart. Data were interpreted using standard run chart rules for improvement projects, wherein the data are examined for shifts, trends, runs and extreme outliers compared with the baseline median.^26^

Qualitative transcripts were analysed by two researchers with a focus on sections relating to QICs, using the framework method.^27^ The interviews were divided into sections about each intervention from the package. Coders reviewed all sections looking for references to QI or for references to how the interventions worked together. Each transcript was dual-coded for consistency in understanding based on a codebook that emerged from a preliminary analysis of the transcripts and according to study objectives. Data were summarised into a framework matrix, which was used to articulate emerging themes and develop the written analysis.

## Results

### Quantitative results

**Figure 2:**
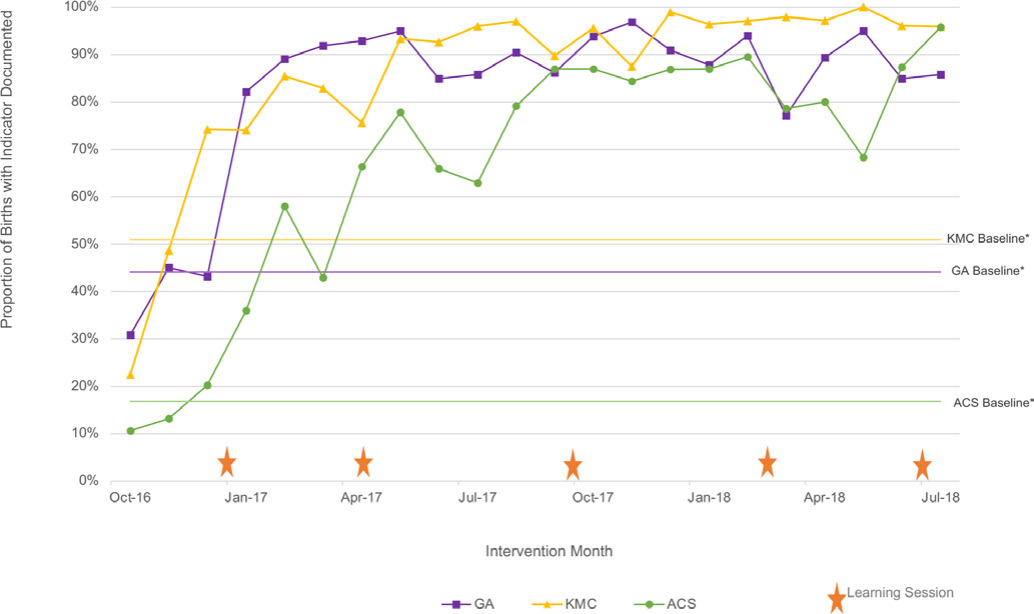
Aggregated run charts, Uganda. *Baseline calculated as the average of the first three values. ACS, antenatal corticosteroid; GA, gestational age; KMC, Kangaroo Mother Care.

**Figure 3:**
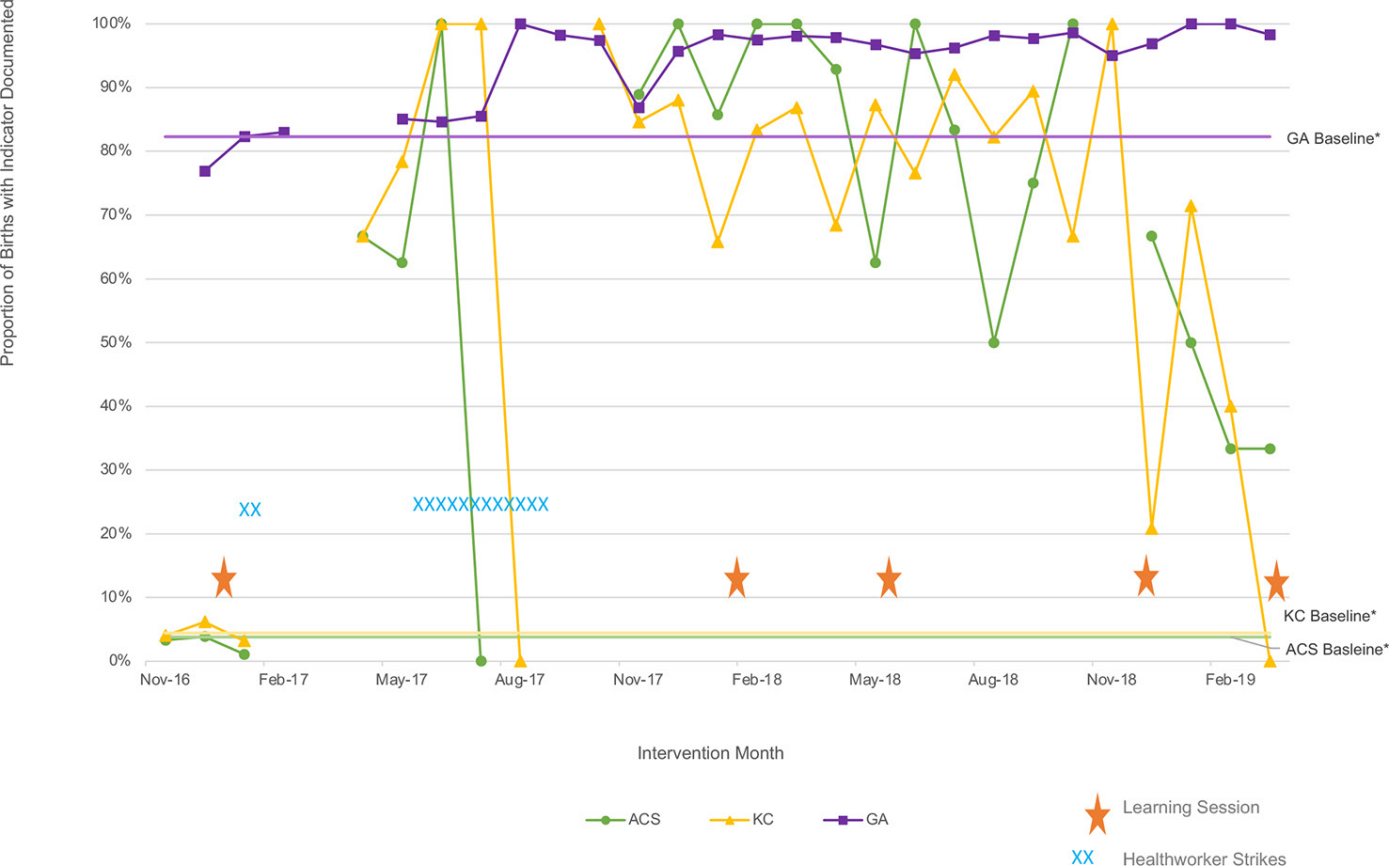
Aggregated run charts, Kenya. *Baseline calculated as the average of the first three values. ACS, antenatal corticosteroid; GA, gestational age; KC, Kangaroo Mother Care.

Aggregated run charts by country are shown in figures 2 and 3. Across both countries, all three of the key shared indicators tracked by each country’s QIC improved using run chart rules.^26^

In Uganda, the recording of GA in the patient charts improved from a baseline median of 44% to a post-intervention median of 86%, appropriate KMC initiation from 51% to 96% and indicated ACS use from 17% to 74%. In Kenya, the pattern was more variable due to health worker strike-related data gaps and work stoppage, but still met improvement criteria.^26^ The recording of GA in the patient charts improved from a baseline median of 82% to a median of 96% by the end of the QIC while appropriate KMC initiation went from 4% to 74% and indicated ACS use improved from 4% to 57%. In Kenya, gains in KMC and ACS were higher in the first year of the QIC but were not sustained, ultimately settling at about 40% and 32% respectively.

### Qualitative results

Qualitative results from IDIs revealed several key themes. Participants appreciated the QI activities and found the data collection and regular meetings beneficial to their ability to develop, test and implement change ideas and specifically benefited from the collaborative nature of the QIC approach. The success of the package integration also came through clearly in the interviews, as some providers were unable to disentangle the benefits of the QIC from the other package components. However, cross-cutting challenges related to availability of supplies and human resources were expressed as barriers to QI practice. Staffing shortages and competing tasks affected providers’ participation in the QI activities. Once solutions were developed and shared across facilities, resource constraints sometimes prevented participants from implementing solutions used in other facilities. This underscored the importance of involving facility and county/district leadership in QI activities.

#### QI cycles engaged providers in data use and interpretation

The PDSA improvement cycle was an impactful and appreciated tool among providers. Collecting data to monitor indicators using QI journals encouraged individuals to learn from and interpret their own data rather than just process it and report it upwards to managers. Shared responsibility for data collection facilitated a more thorough understanding of problems and areas of improvement:

Data collection was something we had given to one person, but from the meetings (we) were able to see that everyone is involved, and we are able to identify gaps in the data as a team and therefore (it has) helped us improve. (Uganda Participant 15)

By handling and recording the QI data, staff felt ownership of indicators and motivation to improve them. Using the collected data at regular meetings facilitated the development of solutions to their discovered problems:

This data was very good because it would show us where we have done very well and also where we are not doing well and we could devise means of how to improve by coming up with change ideas also called possible solutions for the area of improvement. (Uganda Participant 11)

Staff appreciated their interactions with the data because they were responsible for solution implementation, ‘[At my facility, we] see where the data is low then we discuss and come up with a solution for us, because it is us who are going to implement them’*. (*Kenya Participant 5)

Working together with their data enabled teams to identify available resources and make decisions as to how to best address problems. For example, learning sessions in Kenya revealed inadequate room temperature controls, which once documented, was addressed through structural improvements to lighting and insulation. Health facility leadership was also important in this QI process through their verbal support of the programme, encouragement of participation by ‘giving (them) time’ (Uganda Participant 3) to attend and by purchasing of supplies needed to solve identified issues. Leadership’s encouragement of participation was especially important because one of the challenges identified to a successful QIT meeting was low participation, which at times affected teamwork and the success of improvement solutions.

#### QI was more powerful in a collaborative format

The QI intervention was strengthened by the structure of a cross-facility QIC. In multi-facility learning sessions, participants presented on indicators over time, demonstrating learnings and solutions developed during their team meetings. Learning presentation and data analysis skills, as well as teaching others about their solutions, encouraged participants to comprehensively interpret their data over time and whether there had been any improvement, worsening or stagnation.

Friendly competition served as a powerful motivator to generate improvements, as described by Uganda Participant 2, ‘(We were) aiming higher at our performance, if you see that your friends have presented well from other hospitals, you also try towards good performance’. Participants saw learning sessions as feedback where they compared themselves with other facilities. ‘(Learning Sessions were) very useful because we learn from them and…you feel that jealousy you say we are going back to improve’. (Kenya Participant 7) This competitive motivation also encouraged teamwork,

The others elsewhere have been able to implement their work and are performing better than us, I actually say this as just an (opportunity) for us to be determined and work as a team and have that mind of completing the work. (Uganda Participant 4)

While comparison was motivating, participants recognised that improved quality of care within each facility was the ultimate goal. As stated by Kenya Participant 11, ‘it’s not about doing it for the sake of PTBi but to (sic) for the sake of our patients to receive quality care’.

Participants emphasised the increased learning associated with cross-facility communication. Participants reported gaining new knowledge and skills by learning how other facilities solved similar problems. During learning sessions, providers ‘learn how they overcome their challenges with their different hospitals’ (Uganda Participant 9) and ‘identify the gaps and how to fill those gaps’ (Uganda Participant 10). Borrowing and adapting solutions developed by other facilities allowed teams to generate solutions more efficiently, as described by one participant:

What was useful is that we (could) share knowledge and skills from different facilities; (learning) what they do differently that we can copy and also use…we see which (facility) can do better and we go with that (solution). (Uganda Participant 1)

These shared learnings created a more efficient solution development process because rather than starting from scratch, they could choose from methods other teams used and build off their lessons.

#### The importance of package integration

The PTBi QIC was part of a larger intervention package designed to improve quality of care. The integrated nature of this package made it sometimes unclear to participants which aspect of the intervention was being discussed in interviews because they did not see them as separate. The QIC was enhanced by its interaction with the other package components as described by a participant, ‘they complement each other because one has to be there for the other to succeed’. (Kenya Participant 2)

First, the data strengthening component of the package created a more organised and accessible data system, improving health workers’ understanding of and ability to use data from routine sources. This allowed the QITs to better collect and process their own data, and therefore track indicators. Second, since the mSCC served as a data source to track ACS provision, a core QI indicator, completion of this intervention component was directly linked to QI activities, as described by a participant. ‘I was oriented (to the mSCC) by the in-charge but when we started doing QI is when I started understanding its real importance’. (Uganda Participant 13) Lastly, QI mentors and clinical mentors who conducted PRONTO simulation and team training and bedside mentorship were cross-trained and often addressed both areas during facility visits. Thus, providers when commenting on the benefits of mentorship may have been referring to QI, PRONTO or both as illustrated by the following quote ‘Quality improvement has really helped us here and has made our data (better). People used to not document but since we started there is one of the PRONTO people who takes us through quality improvement…and with that we do things the way it is supposed to be done’. (Kenya Participant 4) Mentorship was highly valued by participants as a tool to encourage participation from peers and offered ‘tips on what to do’ (Kenya Participant 1) and advocacy for necessary resources.

This last theme highlights the fact that QI is more than QICs and PDSA cycles. All of the PTBi-EA project components fall under the broader umbrella of QI. As such, while we attempt here to examine the specific impacts of activities related to QICs, the QICs were never intended as stand-alone interventions.

## Discussion

This study provides insight into the impact of the PTBi-EA QI intervention package by examining the QIC component. This component was designed to complement the other interventions and to track process measures of evidence-based practice uptake across facilities. The work of QITs, shared in the QIC, strengthened the impact of the overall project by identifying bottlenecks and roadblocks, creating local solutions, tracking improvements over time and facilitating peer-to-peer learning and sharing of improvement ideas and results. Although it is natural to want to disentangle which elements of our QI intervention package drove the impact observed, all components worked in synergy to improve quality.

Our study demonstrates that evidence-based practice uptake across the three practices tracked in QICs improved in both countries. This pattern is consistent with the study’s primary findings of reduced neonatal mortality and the published theory of change showing improved uptake of evidence-based practices associated with improved outcomes.^21 25^ In Uganda, data reflected a consistent trend with sustained achievements. Kenya’s data reflected a more variable pattern, perhaps due to a greater number of small facilities, data points with denominators of zero and data gaps due to the health worker strike.

Qualitative data demonstrates that the implementation of QICs promoted collaboration across providers and facilities. Staff described increased ownership, skills and motivation gained by processing data, testing change ideas and tracking key metrics, while being supported by QI mentors. Presenting improvements and discussing barriers in cross-facility learning sessions served to magnify learning for providers. The friendly competition derived from cross-facility presentations motivated staff to develop solutions and produce positive results to present back to other teams. This peer-to-peer learning was described as more efficient by building off existing solutions and co-developing ideas across facilities.

Collectively, these data demonstrate that the iterative approach of a QIC supported learning and accelerated improvement to improve uptake of evidence-based practices.

### Interpretation

Similar to a programme in Niger that included a QIC, provider training and posters for healthcare workers, we found that a QIC was successful when implemented with other interventions including provider clinical and team training.^19^ The contribution of the QIC is supported conceptually by the recommendation by the WHO to include ‘learning and sharing’ both within and between facilities, as one of the five functions to improve quality of care for maternal newborn and child health.^28^

Others also have found QI cycles to be a successful intervention, when implemented with clinical training, without using the QIC approach. For example, researchers in Nepal introduced a package of interventions that included facility-based QITs, strengthened leadership accountability and provider clinical training resulting in a reduction in intrapartum-related neonatal mortality.^29^

Contextualised decision-making on whether to use a QIC versus facility-based QITs alone is important. In contexts where QI work is not widespread or institutionalised, QICs may help accelerate uptake of QI practices and spread the culture of QI. Our results support an integrated approach in which QI projects include a QIC component, as advocated in some of the seminal texts for improvement advisors.^30^ As clearly highlighted by the qualitative results, QICs reinforced the other package components, which in turn supported the QIC efforts.

### Study strengths and limitations

This multi-methods analysis demonstrated that QICs are acceptable and feasible to frontline health workers. Further, our approach was successful in two countries with different health facility levels and systems.

However, study limitations exist. Consistent failures in the healthcare system for adequate staffing, timely compensation of staff (resulting in a 6-month nursing strike in Kenya), adequate supplies and equipment may have limited the impact of the QITs, including feasibility of identified solutions. Aggregation of data across facilities reflected the overall success of each country’s QIC, but may conceal variations at facility level. Lastly, our findings are limited by the lack of direct linkage between the quantitative and the qualitative data and the fact that qualitative data in Uganda was collected after the primary study was complete.

## Conclusions

This multi-methods analysis of QIC integration into a larger QI project showed that it was a highly acceptable approach that stimulated improved uptake of evidence-based practices, which in turn may have contributed to health outcome improvements. These findings support the idea that supportive cross-facility peer learning and group problem solving takes place in QICs. Additionally, a multi-pronged approach where several integrated interventions support each other can reinforce good clinical practice from multiple angles. Further research on the cost-effectiveness of these approaches is needed. Policymakers should seek ways to integrate collaborative structures into local and national practice of QI and link efforts to complementary interventions to drive context-relevant change.

## Data Availability

Data are available upon reasonable request. The datasets used and/or analysed during the current study are available from the corresponding author on reasonable request.
