# Supplementary material for: Quality improvement collaboratives as part of a quality improvement intervention package for preterm births at sub-national level in East Africa: a multi-method analysis
Source: BMJ Open Qual. 2023 Dec 22;12(4):e002443. doi: 10.1136/bmjoq-2023-002443 (PMC10749082; doi:10.1136/bmjoq-2023-002443)
Supplement: Supplementary data [file bmjoq-2023-002443supp001.pdf]

SUPPLEMENTARY MATERIAL

Table S1: Change ideas used in Kenya and Uganda Collaboratives

Process to be improved	Effective change ideas
GA determination	1.**Conduct training**<br>CMEs, on-the-job training, bedside mentoring
	2.**Reminder systems**<br>Admissions desk, attach GA wheels to exam room, ward round trolley, and labor suite
	3. **Standardization of care**<br>Triage/verification by qualified midwife, mothers with unknown LMP: ask probing questions, send for ultrasound, systematic orientation for new staff
	4. **Affordances**<br>Ultrasound machine in maternity ward
	5. **Improve the work environment**<br>Tea room for meetings, reports, problem-solving
	6. **Close supervision and feedback**<br>Spot checking for documentation with immediate feedback
ACS (to eligible mothers)	1. **Conduct trainings**<br>Orientation on eligibility and protocols through CMEs and bedside mentoring
	2. **Take care of the basics**<br>Engage administration on procurement and continuous availability, provide space for examination of mothers
	3. **Affordances**<br>Ensure availability at triage desk in emergency tray
	4. **Reminder systems**<br>Visual – pinning up protocols at triage and labor suite, Verbal – checking on needs for ACS during shift handover
	5. **Minimize handover errors**<br>Proper handover during shift change with a written file rather than just verbal
	6. **Reduce controls on the systems**<br>Empower midwives to administer ACS without prescription from doctor
	7. **Use coordinator**<br>Duty allocation of particular midwife to be responsible for ensuring all those eligible for ACS receive it
KMC (for eligible babies)	1. **Standardization of care**<br>Babies in special care unit (SCU) reviewed regularly by a pediatrician (e.g. 3x/week)

	**2. Take care of the basics**<br>Allocate particular midwife to SCU, identify space for mothers doing KMC
	**3. Coach clients to use/products**<br>Practice KMC in SCU before transfer to KMC room, use success stories for first-time mothers to adhere to KMC
	**4. Co-locations**<br>Co-locate SCU and KMC room with labor ward to optimize midwives' time
